# Optimizing temperate pasture-based systems through strategic selection of forage plants

**DOI:** 10.3168/jdsc.2026-1028

**Published:** 2026-02-28

**Authors:** Daniel J. Donaghy, J. Eruera Tait-Jamieson, Keith G. Pembleton, Donita L. Cartmill, Samuel S. Wilson, Michael Egan, Varthani Susruthan, Rebekah L. Wood, Nerissa J. Edwards, Robert C. Southward, Mark A. Osborne, Andrew D. Cartmill

**Affiliations:** 1 School of Agriculture and Environment, Massey University, Palmerston North, 4412, New Zealand; 2 Centre of Sustainable Agricultural Systems, University of Southern Queensland, Toowoomba Queensland, 4350, Australia; 3 Teagasc Agriculture and Food Development Authority, Animal and Grassland Research and Innovation Centre, Moorepark, Fermoy, Cork P61 P302, Ireland; 4 Department of Biosystems Technology, Faculty of Technology, University of Jaffna, Kilinochchi 42400, Sri Lanka; 5 Keenan and Sloan Limited, Agricultural Consultants (KS Agri), Feilding, 4777, New Zealand

## Abstract

This mini-review examines the history and evolution of temperate pasture forage plant selection, from the diverse meadow-inspired mixtures of the 17th century to the simplified perennial ryegrass-based pastures from the early 20th century onward. Climate change, environmental pressures, and market expectations have driven a renewed interest in diverse and alternative pasture systems as a means of enhancing pasture productivity and resilience. However, challenges persist regarding the scale, scope, and transferability of research findings to commercial grazing conditions. We propose a pragmatic, science-based framework that prioritizes functional trait diversity over species richness, guiding farmers to define clear objectives, identify constraints, and select a modest number of complementary species and cultivars based on site-specific conditions and management capacity. This approach recognizes that meaningful functional benefits arise from strategic species and cultivar selection rather than simply increasing species number. We review some existing forage selection tools, noting their value and highlighting their limitations in addressing diverse pastures and holistic environmental objectives. Future advances should integrate resilience traits, ecosystem services, and management considerations into decision-support tools that are co-developed with farmers, researchers, and rural professionals to ensure that biological complexity remains practical and economically viable within pastoral systems.

## Highlights

• Diverse pastures can enhance system productivity and resilience.
• Benefits arise from strategic plant selection rather than increasing plant number.
• Farmers can optimize their pasture systems by using our framework.
• We propose an approach to enhance use of existing pasture selection tools.

## Graphical Abstract

Forage plant selection in temperate pasture systems integrates farmer needs (e.g., seasonal feed supply and soil fertility), location (climate, soil, and topography), and site-specific constraints, alongside clearly defined objectives centered on functional trait diversity and species compatibility, rather than species richness alone. These inputs direct production system design and management decisions, including livestock type, grazing intensity, stocking rate, water availability, and fertilizer use. In turn, these decisions determine the selection of a small number of complementary plant species and cultivars, typically combining grasses, legumes, and herbs. These interactions shape pasture composition and system performance.

**Figure:**
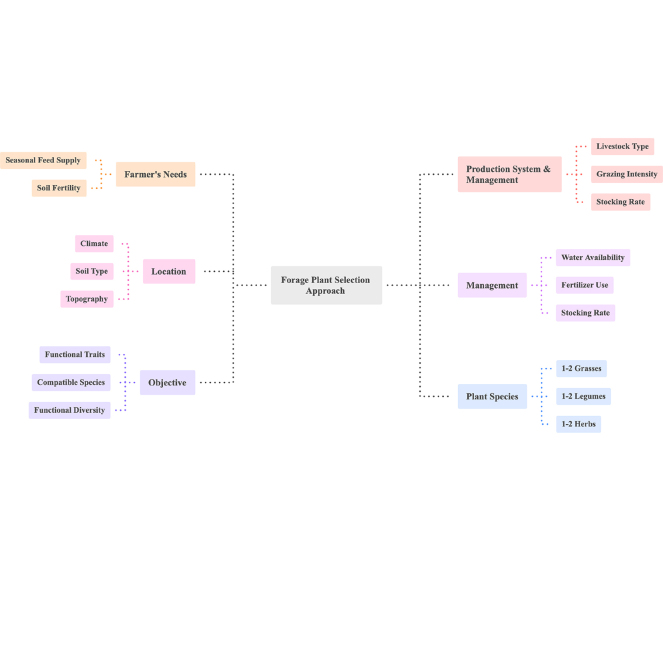

Grasslands cover ~40% of the Earth's terrestrial (nonfrozen) surface (Blair et al., 2014), and livestock production derived from grasslands accounts for ~40% of global agricultural gross domestic product (Salmon et al., 2020). Beyond their productive roles, grasslands are culturally significant and support the livelihoods of >2 billion people worldwide, along with providing a wide range of ecosystems services, including habitat, biodiversity, carbon (C) sequestration, and water regulation (Bengtsson et al., 2019). This mini-review aims to stimulate discussion around selection of the most appropriate forage species and cultivars for temperate pastoral systems.

In the mid-1600s, efforts to improve grasslands across Europe aimed to emulate native meadows by harvesting and sowing seed mixtures comprising multiple productive species (reported by Gilliland, 2022). During the 1700s, this approach changed toward simpler species combinations, typically mixtures of ryegrasses (*Lolium* spp*.*), white clover (*Trifolium repens* L.), and bird's-foot trefoil (*Lotus corniculatus* L.), to align with arable crop rotations and emerging mixed-farming systems (Charlton, 1991). By the late 1800s, the focus again switched back to pursuing greater pasture persistence by sowing multispecies (“diverse”) pastures (Stapledon and Davies, 1948). The intensification of agriculture in the 1930s resulted in renewed preference worldwide for simplified pasture mixtures, generally based on a binary mixture of one grass and a companion legume (Levy, 1933). Commonly sown grasses included perennial ryegrass (*Lolium perenne* L.), tall fescue (*Lolium arundinaceum* [Schreb.] Darbysh.), and orchardgrass (syn. cocksfoot; *Dactylis glomerata* L.), and accompanying perennial legumes included white clover, alfalfa (syn. lucerne; *Medicago sativa* L.), and red clover (*Trifolium pratense* L.). Perennial ryegrass emerged as the dominant temperate pasture species globally, frequently paired with white clover (Frame and Laidlaw, 2011). These simple mixtures established rapidly, produced high-quality herbage suitable for intensive pastoral livestock systems, and were easier to manage than the more diverse mixtures sown previously (Kemp et al., 2000). In addition, perennial ryegrass-white clover pastures proved adaptable to a wide range of management practices, including both rotational grazing and set stocking (Brock and Hay, 1996).

From the 1950s onward, increasing availability and affordability of synthetic nitrogen (N) fertilizers resulted in their increased usage to stimulate pasture yield and reduce seasonal variability. In several temperate pastoral regions, including Ireland and South Africa, this prompted the gradual removal of the legume component from sown pasture mixtures, resulting in N-fertilized monocultures, typically ryegrasses (McNamara, 2019; Phohlo et al., 2022). In contrast, in other regions, including New Zealand and Australia, the legume component generally remained as an integral component of sown pastures, although reliance on N fertilizers steadily increased (Rawnsley et al., 2019; Gray, 2024).

In recent decades, climate change has negatively affected the persistence and productivity of perennial ryegrass (Lee et al., 2013; Wang et al., 2024), and this, along with shifting market expectations of greater biodiversity in agricultural systems and public scrutiny of the environmental footprint of pastoral agriculture are necessitating a reevaluation of agricultural production and resource use, including GHG mitigation, lower nutrient inputs and losses, and enhanced system resilience (Reheul et al., 2017; Thomson and Albornoz, 2023; Finn et al., 2024). In response, pastoral farmers and researchers are reexploring alternative forage species and management practices. For example, the use of alternative grasses to perennial ryegrass has been reported to buffer system response to climate volatility (Rogers et al., 2022), incorporating legumes into grass-dominant pastures has been revisited as a means to reduce reliance on synthetic N fertilizers (Egan et al., 2018), and the inclusion of plantain (*Plantago lanceolata* L.) has been promoted as a mitigation strategy to reduce N losses from pastoral systems (Pinxterhuis et al., 2024). In addition to these incremental adjustments, there is again renewed advocacy for the use of more diverse pasture mixtures, as an adaptive response to climate change (Reich et al., 2001), to improve a range of desired ecosystem functions (Suter et al., 2021; Finn et al., 2024), and as part of regenerative agriculture (Jordon et al., 2022).

Modern diverse pasture mixtures are generally short- to medium-term (lasting 4–6 yr) and comprise forage species with complementary functional traits (i.e., grass, legume, and broadleaf or herb) and high nutritional value for grazing livestock (Jaramillo et al., 2021; Thomson and Albornoz, 2023). The total number of species in these diverse pasture mixtures generally ranges from 3 to 12 (Jaramillo et al., 2021).

Reported benefits of more diverse pasture mixtures include: improved resilience to climate volatility (Sanderson et al., 2005; Skinner and Dell, 2016; Lüscher et al., 2022), increased or greater stability in annual yield (Sanderson et al., 2007; Jaramillo et al., 2021; Jordon et al., 2022), enhanced animal production and performance (Chapman et al., 2008; Cranston et al., 2015; Sheridan et al., 2022; Beaucarne et al., 2025), enriched ecosystem diversity above and belowground (Buzhdygan et al., 2020; Cong et al., 2020), along with environmental benefits including reduced N losses (Beukes et al., 2014; Pembleton et al., 2015; Bracken et al., 2022), reduced GHG production (Boland et al., 2022; Bracken et al., 2022), increased soil C levels (Skinner and Dell, 2016; Buzhdygan et al., 2020; Jordon et al., 2022), and increased soil structure and porosity (Gould et al., 2016).

However, there are multiple challenges with the size, scale, duration, and transferability of some of these studies. For example, some are smaller scale, mown plot studies, and because pasture diversity generally remains higher and more stable under mowing than grazing (Thomson and Albornoz, 2023), these results are not easily extrapolated to grazing conditions. Many studies also used a grass monoculture as their control, which again is not representative of many pastoral systems (Star and Brooking, 2007; Boland et al., 2022; Sheridan et al., 2022). Also, despite their initial productivity, many high-yielding diverse pasture mixtures were relatively short-term, with nearly half of the sown species not persisting beyond 3 or 4 years (Sanderson et al., 2007). Multiple studies have reported that within several years, pasture mixtures reverted to grass dominance, typically driven by the competitive advantage of fast-growing grass species under fertilized conditions (Tracy and Sanderson, 2000; Sanderson et al., 2007; Scott et al., 2018). Finally, Finn et al. (2024) cautioned against directly transferring insights from studies on native or semi-natural grasslands, which are typically characterized by higher species richness, minimal or no fertilizer inputs, lower yields, and infrequent harvesting, to intensively managed, high-productivity pastoral systems.

This brief review of historical recommendations and trends in pasture species selection shows the somewhat cyclical pattern whereby, for different reasons, simple and diverse mixtures have been promoted, and highlights a need for clear information that allows farmers to make informed decisions that are relevant for their circumstances and location. The remainder of this paper outlines a pragmatic, science-based approach that is farmer-centric, and promotes the selection of the most appropriate forage species and cultivars that meet farmers' requirements and are best suited for their location and management. This approach (Figure 1) is broadly comparable to that outlined by Hobbs and Morton (1999), which mimicked natural ecosystems.

**Figure 1:**
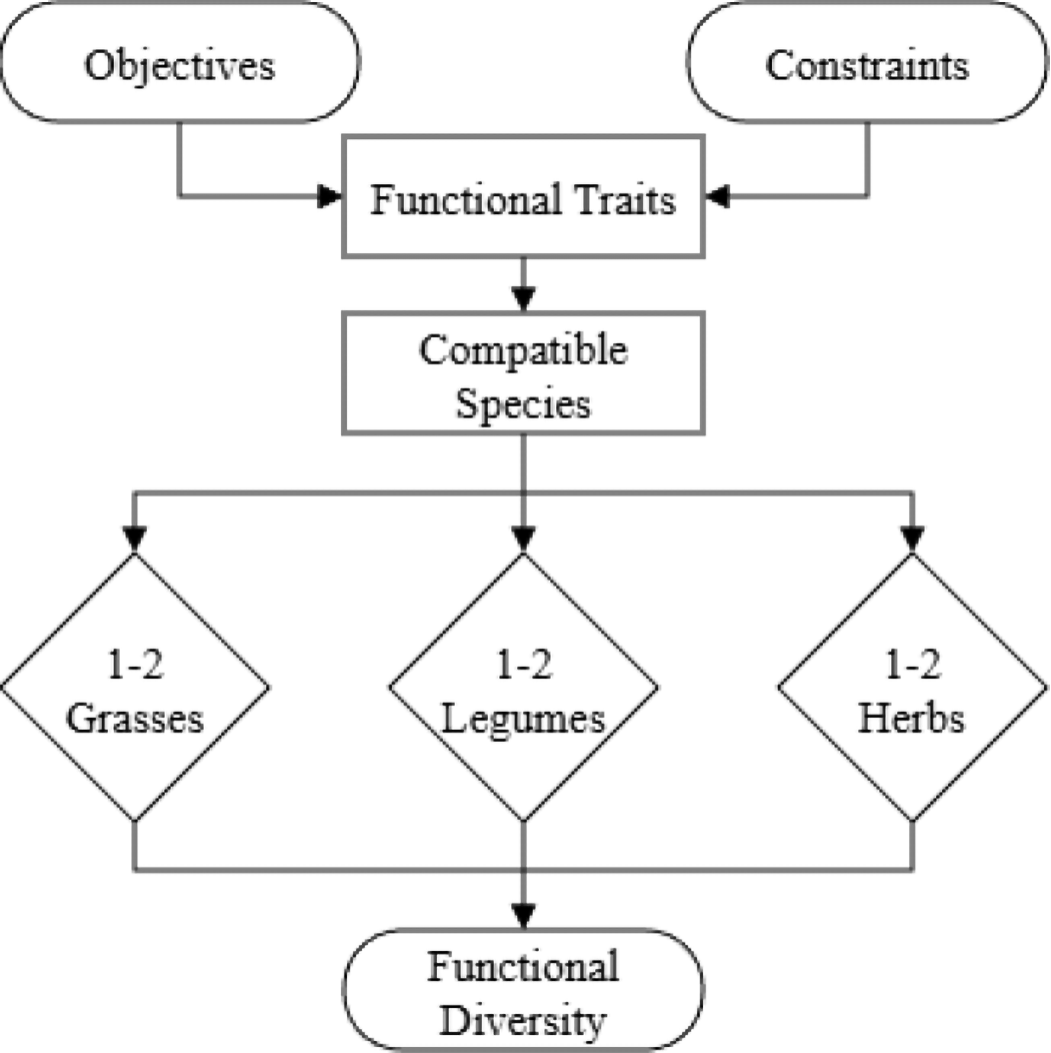
An approach to aid farmers and rural professionals in selecting combinations of forage plant species that match the farmers' needs and production system, and suit their location and management.

Functional diversity (Figure 1) represents the range and significance of functional traits in pastoral systems (Tilman et al., 1997; Díaz and Cabido, 2001), including leaf size, canopy height, N fixation, and rooting depth (Díaz and Cabido, 2001). Functional diversity is important because it is a key determinant of multiple factors, including plant productivity, plant N content, and light penetration into a canopy (Tilman et al., 1997). Functional diversity of a pasture, rather than the total number of individual species present, strongly affects ecosystem functioning (Tilman et al., 1997; Díaz and Cabido, 2001), which includes processes such as production, nutrient cycling, and water dynamics (Díaz and Cabido, 2001).

The first step in designing an effective pasture system is for farmers and rural professionals to clearly define their objectives, in terms of what they want their pastures to achieve. Traditionally, farmers and rural professionals have focused on objectives such as a more even pattern of annual production, improved seasonal growth, and increased animal performance (Rawnsley et al., 2013). However, in more recent years, additional objectives have emerged that reflect changing climate and environmental pressures. These include denser ground cover and increased persistence during summer droughts (Tilman and Downing, 1994; Rogers et al., 2022), greater resilience to flooding or waterlogging (Brotherton and Joyce, 2015; Oram et al., 2021), reduced use of N fertilizer inputs (Baker et al., 2023; Jezequel et al., 2024), reduced losses of N and other nutrients (Malcolm et al., 2014; Vibart et al., 2016; Schon et al., 2024), and deeper rooting to enhance soil health, nutrient cycling, and drought tolerance (Eisenhauer et al., 2011; Jordon et al., 2022).

We suggest that these objectives should be defined as clearly and specifically as possible. For example, the objective of improving drought tolerance is vague, whereas specifying a target such as maintaining >50% ground cover during summer dry periods provides a tangible and measurable outcome that can guide decision making.

In parallel with clearly defining the objectives, farmers and rural professionals should also identify key constraints, by assessing whether pasture species and cultivar selection is the key limitation or opportunity. We suggest that in many cases, underlying factors including farm layout (e.g., an excessive number of small or oddly shaped paddocks that constrain pasture allocation; Maher et al., 2023), suboptimal grazing management (e.g., inappropriate grazing rotation or residual; Donaghy et al., 2021), and inadequate soil fertility and drainage may be primary constraints. We argue that introducing new forage species will not resolve these underlying constraints and may lead to disappointing establishment or persistence if these constraints are not addressed first (Cartmill et al., 2025).

Farms are rarely homogeneous; for example, within a single property, variations in soil type, slope, aspect, and drainage can create distinct microenvironments, thereby presenting individually or in combination different opportunities and limitations. Furthermore, farms typically serve different operational roles, each with unique management priorities and pasture performance expectations (e.g., milking platform, cropping zones, or grazing areas for young or dry stock). Last, location-specific weather conditions can further create micro-climates that influence both the establishment and persistence of specific pasture species.

For these reasons, some farmers already match different pasture types to different land classes or functional roles within the farm system. For example, perennial ryegrass-white clover pastures are often established on flatter, more fertile areas suited to both grazing and conservation, whereas legume-herb pastures are commonly used to fatten young stock. This context-based approach, where the attributes and constraints of a farm system are considered, enables the targeted allocation of forage species or pasture mixtures to areas where they are more likely to perform well. Consequently, each pasture type can be managed in accordance with its specific ecological requirements and production potential, thereby improving both system efficiency and resilience.

Once the objectives are clearly defined and key constraints identified, the next step is to select forage species or cultivars with functional traits that align with those objectives and address the identified constraints. Many resources are available to support this, including seed company websites, guides and booklets; agronomic extension materials; and digital forage selection indices and other online tools (discussed later in more detail). For example, if the objective is to fill a summer feed gap, selecting deep-rooted species such as lucerne, chicory (*Cichorium intybus* L.), or tall fescue may be appropriate (Hayes et al., 2010; Perera et al., 2019; Churchill et al., 2022). Conversely, if the goal is to improve plant performance under conditions of poor drainage or periodic waterlogging, species such as tall fescue, timothy (*Phleum pratense* L.), Kentucky bluegrass (*Poa pratensis* L.), and strawberry clover (*Trifolium fragiferum* L.) may be considered (Wang and Jiang, 2007; Striker and Colmer, 2017; Oram et al., 2021; Moreno et al., 2023; Smith et al., 2023).

Critically, the benefits of increased pasture diversity depend not on simply adding more species into a mixture, but on the strategic selection of forage plant species that deliver complementary functions to the pastoral system (Sanderson et al., 2007; Pembleton et al., 2015; Thomson and Albornoz, 2023; Finn et al., 2024). The compatibility of these species is therefore another key step (Suter et al., 2021; Finn et al., 2024). Each selected species should occupy a distinct ecological niche, for example, a different growth pattern, phenology, or resource use, and thereby contribute to pasture system performance by either fulfilling unique functions, performing different functions, or performing similar functions at different times of the year (Cardinale et al., 2007; Sanderson et al., 2007).

Conversely, when 2 or more species share similar growth dynamics and environmental requirements, direct competition occurs as they compete for the same limiting resources, leading one species to dominate while the other declines, ultimately conferring little to no functional advantage to the system. However, native grasslands often sustain high species richness because plants differ subtly in traits such as rooting depth, seasonality of growth, and nutrient uptake strategy, thereby allowing niche differentiation to reduce direct competition and exclusion. The functional redundancy hypothesis complements this by proposing that many grassland species perform similar ecological roles (e.g., N uptake and C sequestration), and so the loss of one species may not immediately impair ecosystem function (Rodríguez et al., 2015; Kalmykov and Kalmykov, 2016; Hening and Nguyen, 2020).

Working through these processes of clearly defining the objectives along with the constraints within the farm system will generate a list of forage plant species that are both functionally relevant and compatible with specific conditions on the farm. We argue that to achieve meaningful functional diversity, a pragmatic approach would involve selecting 1 or 2 forage species from 2 or 3 functional groups, resulting in a total pasture seed mixture of ~2 to 6 species or cultivars. Consistent with Finn et al. (2024), we contend that pasture mixtures should be designed around a targeted selection of high-performing species, rather than assembled randomly, to maximize system performance with a limited number of species.

The scientific rationale for this recommendation is supported by studies demonstrating that mixing grasses, legumes, and herbs in various combinations provides a range of benefits, both over monocultures and in some cases, binary mixtures of perennial ryegrass-white clover, including total yield, yield stability, nutrient cycling, and weed suppression, and this is typically achieved with between 4 and 8 species (Suter et al., 2021; Tilman et al., 2001; Lüscher et al., 2022; Finn et al., 2024; O'Malley et al., 2026), with between 3 and 5 species dominating at any given time (Tilman et al., 2001). Similarly, most reported improvements in animal performance between simple and diverse pastures were achieved with mixtures of just 3 or 4 species (Jordon et al., 2022). Increasing the number of species beyond this range was generally not reported to provide additional functional or production benefits. In this context, diversity should be considered in terms of functional roles rather than simply the number of forage plant species or cultivars included in a pasture mixture (Sanderson et al., 2007; Finn et al., 2024). We argue that this principle has a clear biological basis, where each species must achieve a sufficient population density, either in plant numbers or in tillers or growing points, in order to effectively perform its intended function. At the same time, finite resources within a sown area, including light, water, and nutrients, constrain total yield. Consequently, distributing these limited resources among too many species may reduce the likelihood that any single species achieves a population size that can deliver measurable functional benefits (Bengtsson et al., 1994; Tilman and Downing, 1994; Adler et al., 2013; Thibaut and Connolly, 2013; Isbell et al., 2018; Finn et al., 2024).

Functional diversity may also be enhanced without increasing the number of species, by exploiting genetic and phenotypic variation among cultivars within key forage species. For example, perennial ryegrass cultivars differ markedly in flowering time (and therefore annual yield distribution), seasonal growth activity, and growth habit (e.g., tetraploids tend to be taller compared with diploids; Frame and Boyd, 1986). Similar genotypic differences occur in white clover, where small-leaved cultivars are better suited to set-stocked sheep systems, whereas large-leaved cultivars are better suited to rotational grazing by cattle (Caradus et al., 1996). Therefore, it is possible for a farmer growing a traditional perennial ryegrass-white clover pasture to increase functional diversity simply by selecting cultivars from within both of those species. Pollnac et al. (2014) demonstrated that mixtures of 6 perennial ryegrass cultivars produced higher yields with increasing cultivar diversity, with the greatest yield increase observed in combinations that balanced traits such as winter hardiness, heading date, and grazing tolerance. However, other studies have reported limited effects; for example, Culleton et al., 1986, Dairy NZ, 2024 and Gilliland et al. (2011) both reported no yield increase from mixing perennial ryegrass cultivars, although both studies observed improved yield stability from the mixtures over 3 to 4 years. Gilliland et al. (2011) cautioned that increasing the number of cultivars in a mixture may not replicate the synergistic effects observed in diverse pastures.

Farmers and rural professionals have access to a wide range of resources to support selection of forage plant species, and examples include the Value Indices in New Zealand and Australia (**NZFVI** and **AUFVI**, respectively), the Irish Pasture Profit Index (**PPI**), various USDA Cover Crop Species Selectors, and the Swiss List of Recommended Forage Crop Varieties. The application and limitations of these tools will be briefly reviewed, followed by a discussion of the implications for forage species selection and implementation.

The NZFVI was launched in 2012 to evaluate and categorize perennial and short-term ryegrass, including annual ryegrass (*Lolium multiflorum* L.), Italian ryegrass (*Lolium multiflorum* L.), and hybrid ryegrass (*Lolium* ×*boucheanum*, syn. *Lolium hybridum*) cultivars, using a 5-star rating system. Initially, the ratings were primarily based on seasonal DM yield, with later iterations incorporating ME and persistence (Ludemann et al., 2018). Higher star ratings indicate cultivars that could potentially deliver greater economic returns, derived from linking trait performance data to region-specific economic values (Chapman et al., 2017). However, in early 2024, the NZFVI was suspended pending validation of the predicted economic differences among cultivars (DairyNZ, 2024).

The PPI was launched in 2014 as an economic selection index to identify superior perennial ryegrass cultivars (McEvoy et al., 2011; O'Donovan et al., 2017), and similar to the NZFVI, is derived using data from national evaluation trials, with cultivar performance expressed as a total merit index and then converted into an estimated profit per hectare per year (O'Donovan et al., 2017). Key traits include annual and seasonal DM production, herbage quality in spring and summer, silage DM yield, and pasture persistence (McEvoy et al., 2011). More recently, a grazing utilization trait was added to reflect pasture intake efficiency under rotational grazing (Tubritt et al., 2021).

The AUFVI was introduced in 2017, building on the NZFVI and the PPI, and was initially developed as a perennial ryegrass index based on the economic value of seasonal and annual DM production within 4 major Australian dairy regions (Leddin et al., 2018). Recent expansion included an additional 3 dairy regions, along with annual and Italian ryegrasses (Dairy Australia, 2024), the inclusion of ME, and the simplification of the economic valuation framework (Lewis et al., 2024).

A key limitation of all 3 indices is their strong orientation toward the dairy industry and the focus on perennial ryegrass, with cultivar performance largely evaluated under mown monoculture conditions. None of these resources currently accommodate diverse pastures, although research is underway in Ireland to develop a white clover economic selection index (Carroll et al., 2025), and in Australia, Meat and Livestock Australia's Pasture Trials Network eTool (https://ptntool.mla.com.au/) can identify species other than ryegrass better suited to climate extremes and regional adaptation (Rogers et al., 2022). In New Zealand, hill-country pastoral systems have benefited from resources that combine environmental gradients and grazing impacts to predict the success of pasture mixtures (Scott et al., 1995).

Despite these limitations, all 3 indices provide farmers with access to rigorous validated frameworks of independently collected data (McEvoy et al., 2011). Research indicates that mixture performance can be reasonably accurately predicted by averaging the trait values of component varieties (Griffith et al., 2016), suggesting practical opportunities for farmers to customize seed mixtures that balance complementary strengths.

The USDA has developed and supported several regional decision tools in collaboration with groups such as the Midwest Cover Crops Council (Bladen, NE). There is also a standalone USDA Agricultural Research Service educational resource called the Cover Crop Selector (https://covercrop-selector.org/). Users first select their state and USDA hardiness zone, then apply filters based on desired traits, such as drought or salinity tolerance, ease of establishment, or root depth. The tool then generates a list of recommended species, accompanied by information on sowing rate, potential DM yield, and management considerations. We feel that a major strength of the Cover Crop Selector is its goal-oriented, user-driven design, which allows farmers to tailor forage choices to a holistic range of objectives, including erosion control, N fixation, and soil health improvement.

We suggest that this flexibility supports on-farm experimentation and adaptive learning, helping users deepen their understanding of ecological interactions within their own farming systems. However, we feel that this tool also has several limitations, including the uneven availability of empirical data, and not being tailored for permanent pasture management or grazing system design. Additionally, although the tool provides accessible summaries, it does not currently integrate economic or ecosystem-service modeling.

As our last example, Switzerland has a structured, regionally adaptive approach to forage species selection, where recommended pasture mixtures are determined according to soil, climate and environmental factors, and are tested across multiple locations (Sanderson et al., 2007). These results are summarized in a guide that provides agronomic performance from comparative studies of the commercially available pasture cultivars, along with information on recommended pasture mixtures tailored to a range of environmental conditions and production goals (Suter et al., 2025).

Once the farmer has consulted regional resources such as those listed above and selected the most appropriate forage species or cultivar combinations, they should finally consider their own management capacity and preference. A key consideration is that although the introduction of new or unfamiliar forage species or mixtures may enhance herbage quality and availability and improve systems resilience, as a trade-off, it may also require modification of current management practices (e.g., grazing residual, rotation length, and fertilizer rate and type; Pembleton et al., 2015; Gilliland, 2022; Jordon et al., 2022), which may increase farm system complexity.

As previously mentioned, monocultures or simple mixtures are often favored for their ease of management and predictability. By contrast, the inclusion of additional species introduces both opportunities and trade-offs. For example, cocksfoot and bromes (*Bromus* spp.) both benefit from longer rotations (e.g., 4- to 5-leaf stage; Turner et al., 2006a,b) compared with perennial ryegrass (2- to 3-leaf stage; Fulkerson and Donaghy 2001). Mixing these species may mean compromising optimal management for one or more species. However, innovative grazing systems are emerging that synchronize management across pasture species with different growth dynamics. For example, Oliveira et al. (2026) proposed a mixed pasture of perennial ryegrass, cocksfoot, pasture brome (*Bromus valdivianus* Phil.), and white clover managed on a rotation aligned with the 3.5- to 4-leaf regrowth stage of the brome component. Over 2 years, this approach achieved growth asynchrony between all species and optimized total yield, demonstrating that temporal coordination can reconcile plant species-to-species differences.

In addition, pasture structure and canopy height influence intake by grazing animals and therefore pasture utilization. The optimum postgrazing height for intake by lambs is 60 mm for perennial ryegrass, but 80 mm for chicory and red clover (Kemp et al., 2010), meaning that pasture mixtures containing all 3 species will always theoretically experience some form of overgrazing or undergrazing of individual components when aiming to maximize lamb growth. Seasonal growth patterns also differ; for example, plantain, chicory, red clover, and white clover, which are sown together to improve liveweight gain in lambs, are all dormant or semi-dormant during cold winters (Kemp et al., 2010). This can create a feed gap on farms over the cooler months, and to overcome this, Kemp et al. (2010) suggested sowing up to 20% of the farm to more winter-active species such as Italian ryegrass to compensate for a similar area (between 10% and 20%) sown to the legume-herb mix.

Legume and herb persistence are also affected by grazing management, for example, more erect legumes such as red clover and lucerne are generally less plastic in their response to grazing than white clover, and they benefit from both longer rotations and longer postgrazing residuals to maintain persistence (Moot et al., 2003; Black et al., 2009; Kemp et al., 2010). Similarly, the persistence of herbs benefits from lower stocking rates (O'Connor 2005) and longer postgrazing residuals, along with longer grazing rotations and avoiding treading damage in winter (Ayala et al., 2011). However, these managements can favor companion grasses, which may outcompete herbs and accelerate their decline (Wilson et al., 2024).

Differences in soil fertility and establishment requirements may further complicate management of diverse pastures. For example, lucerne requires soil pH above 6.5, whereas other pasture species (e.g., annual ryegrass and subterranean clover [*Trifolium subterraneum* L.]) tolerate or even prefer more acidic conditions (Scott et al., 2000). Establishment depth is also critical, and many herbs and legume species have small seeds that require shallow seed placement for successful germination (Thom et al., 2011).

More complexity in the forage plant species mixture can create within-paddock management conflicts and increase the risk of management mistakes. To balance these factors, we suggest that farmers develop specialized paddocks that target different functions, rather than combining all selected species into a single mixture. This approach enables the farmer to exploit species-specific advantages (e.g., summer-active herbs or deep-rooted legumes) without compromising the manageability of the overall system. In addition, we suggest that selecting pasture species that complement one another in growth pattern and habit, and that are unlikely to compete directly for resources, can reduce management trade-offs and enhance both productivity and resilience at the whole-farm scale.

Looking forward to functionally integrated, adaptive, and resilient pastures, although technical information to guide species selection is widely accessible, we feel that the true challenge lies in incorporating diversity into current farm systems and management. As the complexity of the pasture increases, so too does the need for adaptive management frameworks that consider both biophysical interactions (e.g., competition, complementarity, and seasonal asynchrony) and managerial capacity. A farmer's ability to align grazing management, nutrient supply, and environmental stewardship with the biological needs of multiple forage species will determine whether diversity translates into greater resilience or simply greater complexity (Pembleton et al., 2015).

We suggest that future advances in pasture species selection and diversity will depend on integrating empirical testing with digital decision-support tools that are practical, yet able to simulate the interactions between species traits, management timing, and environmental variables. Emerging approaches such as functional trait-based modeling; remote sensing of pasture yield, composition, and quality; and machine learning for grazing optimization, offer significant opportunities for pasture species selection and management. We feel that continued innovation in the agronomic sector will make it possible for prescriptive pasture species mixtures to be tailored to site conditions and farmer objectives.

Last, there is also substantial opportunity for the development of region-specific guidelines and indices that go beyond productivity metrics to include resilience, persistence, and ecosystem-service contributions. This would represent an evolution from static recommended lists toward adaptive, data-driven systems that integrate real-time feedback from research farms, on-farm sensors, and farmer networks. Importantly, these future frameworks should be co-developed with farmers, rural professionals, and researchers, in order to ensure that increasing biological complexity remains accessible (including ease of use) and compatible with on-farm operations and long-term economic viability.
